# Treatment of Flat-Foot

**Published:** 1891-01-10

**Authors:** 


### TREATMENT OF FLAT-FOOT.

Trendelenburg has recently treated three cases of congenital flat-foot and one of traumatic origin, resulting from fracture of the ankle-joint, by supramalleolar osteotomy, on the principle of restoring the static equilibrium to which Volkmann attached such importance. Just above each malleolus he makes a horizontal incision through the skin and down to the bone about one cm. in length. With a heavy sharp chisel, made entirely of steel, he works through these incisions on each side alternately, in a transverse or slightly downward oblique direction, until both the malleoli are detached. Holding the ankle under his arm and grasping the foot in the other hand, he presses it firmly, and adjusts it with the utmost accuracy in the normal position, in which it is fixed by a plaster bandage, which should be on the pattern of the "Bavarian boot," the application of which assists the re-position, which a roller might disturb. After ten or twelve days the bandage is removed to give an opportunity for a final readjustment of the foot, while union is still incomplete, for if this re-position be not absolutely accurate, the deformity will return. After four or six weeks the fracture will have become consolidated, and the patient may be allowed to walk, though not without mechanical support in the form of a boot, laced down nearly to the toes, with lateral steel splints, and transverse straps above the ankle.

